# Reclassification of *Enterobacter* sp. FY-07 as *Kosakonia oryzendophytica* FY-07 and Its Potential to Promote Plant Growth

**DOI:** 10.3390/microorganisms10030575

**Published:** 2022-03-06

**Authors:** Ge Gao, Yan Zhang, Shaofang Niu, Yu Chen, Shaojing Wang, Nusratgul Anwar, Shuai Chen, Guoqiang Li, Ting Ma

**Affiliations:** 1 Key Laboratory of Molecular Microbiology and Technology, College of Life Sciences, Nankai University, Ministry of Education, Tianjin 300071, China; 2120150875@mail.nankai.edu.cn (G.G.); 2120201137@mail.nankai.edu.cn (Y.Z.); niushaofang000@163.com (S.N.); 1120160357@mail.nankai.edu.cn (Y.C.); 1120200495@mail.nankai.edu.cn (S.W.); nusrat628@163.com (N.A.); sky_csdo@163.com (S.C.); 2 Tianjin Engineering Technology Center of Green Manufacturing Biobased Materials, Tianjin 300071, China

**Keywords:** *Enterobacter*, *Kosakonia*, reclassification, FY-07, promote plant growth

## Abstract

Precise classification of bacteria facilitates prediction of their ecological niche. The genus *Enterobacter* includes pathogens of plants and animals but also beneficial bacteria that may require reclassification. Here, we propose reclassification of *Enterobacter* FY-07 (FY-07), a strain that has many plant-growth-promoting traits and produces bacterial cellulose (BC), to the *Kosakonia* genera. To re-examine the taxonomic position of FY-07, a polyphasic approach including 16S rRNA gene sequence analysis, ATP synthase β subunit (*atpD*) gene sequence analysis, DNA gyrase (*gyrB*) gene sequence analysis, initiation translation factor 2 (*infB*) gene sequence analysis, RNA polymerase β subunit (*rpoB*) gene sequence analysis, determination of DNA G + C content, average nucleotide identity based on BLAST, in silico DNA–DNA hybridization and analysis of phenotypic features was applied. This polyphasic analysis suggested that *Enterobacter* sp. FY-07 should be reclassified as *Kosakonia oryzendophytica* FY-07. In addition, the potential of FY-07 to promote plant growth was also investigated by detecting related traits and the colonization of FY-07 in rice roots.

## 1. Introduction

The taxonomy of *Enterobacter* has a long and very confusing history, and many species transfers have occurred [1]. Many studies have shown that due to the low phylogenetic resolution of 16S rRNA gene, it is difficult to classify a species into the genus *Enterobacter* only by analyzing the 16S rRNA gene sequence [2]. Brady et al. reclassified the genus *Enterobacter* into several genera including *Kosakonia* through multilocus sequence analysis (MLSA) [3]. According to the LPSN (List of Prokaryotic names with Standing in Nomenclature database) (available online: https://lpsn.dsmz.de/genus/kosakonia accessed on 21 December 2021), so far, there have been nine species in the genus *Kosakonia*: *Kosakonia arachidis*, *Kosakonia cowanii*, *Kosakonia oryzae*, *Kosakonia oryziphila*, *Kosakonia oryzendophytica*, *Kosakonia pseudosacchari*, *Kosakonia radicincitans*, *Kosakonia sacchari* and *Kosakonia quasisacchari* [1,4,5,6,7]. Most *Kosakonia* strains are isolated from commercial crops including maize, rice, sugarcane, cotton, wheat, and have been found to promote the growth of host plants [8,9]. The effect of these strains on promoting plant growth is attributed to the strain’s ability to biologically fix atmospheric nitrogen, dissolve rock phosphates and secrete plant growth hormones [10].

*Enterobacter* sp. FY-07 (CGMCC No.: 6103) was isolated from the oilfield produced water of China Petroleum Natural Gas Co., Ltd. at Jilin Oil Field Branch, Jilin, China, in 2010 [11]. The strain FY-07 was classified as an unclassified species of *Enterobacter* and named as *Enterobacter* sp. FY-07 at that time, because the genus *Enterobacter* was polyphyletic yet the phylogenetic resolution of 16S rRNA gene was low [3]. With the development of the taxonomy of *Enterobacter*, the genus *Kosakonia* was separated from the genus *Enterobacter* [1]. Thanks to its excellent BC production capacity and unique fermentation characteristics, FY-07 has been continuously researched and developed [11,12,13,14,15]. Comparative genomic analysis of a report suggested that FY-07 may be closely related to *Kosakonia* [16]. 

In this study, the classification status of FY-07 was re-examined based on the 16S rRNA gene sequence similarity, MLSA based on the concatenated partial *atpD*, *gyrB*, *infB* and *rpoB* gene sequence, isDDH (in silico DNA–DNA hybridization), ANI (average nucleotide identity based on BLAST), difference in the content of guanine–cytosine (G + C) and biochemical characteristics, including traits associated with plant growth promotion. Additionally, its potential to promote plant growth was also investigated by measuring its ability of IAA (Indoleacetic acid) production, siderophore production, ammonia production, solubilization for insoluble phosphate and potassium, nitrogen fixation, 1-aminocyclopropane-1-carboxylic acid (ACC) deaminase production.

## 2. Materials and Methods

### 2.1. Materials

The strain *Enterobacter* sp. FY-07 (CGMCC No. 6103) was aerobically cultured at 30 °C on Luria–Bertani medium (pH 7.0) composed of 0.5% yeast extract, 1% peptone, and 0.5% NaCl. The rice variety used was *Oryzasativa* spp. Japonica, L. All chemicals were analytical grade unless otherwise stated.

### 2.2. Sequences Acquisition and Phylogenetic Analysis

All 16S rRNA gene sequences, whole-genome sequences and MLSA-related genes sequences were retrieved from GenBank, and all the accession numbers were provided here (Tables S1–S3).

The 16S rRNA sequence (1530 bp) of FY-07 was extracted from the whole genome (CP012487.1). The obtained 16S rRNA sequence was compared on the EzTaxon-e server (available online: http://eztaxon-e.ezbiocloud.net accessed on 4 January 2022) [17]. The amplification and sequencing of protein coding genes *atpD*, *gyrB*, *infB* and *rpoB* referred to the primers and schemes of Brady et al. [1]. The PCR product was purified and recovered using a DNA gel recovery kit (TIANGEN, Beijing, China) and then subjected to Sanger sequencing (GENEWIZ, China). The sequences of related strains obtained from the GenBank database were aligned with CLUSTAL W, and the phylogenetic trees were reconstructed with the neighbor-joining method of the maximum compound likelihood model. Bootstrap analysis was performed based on 1000 repetitions. The MEGA 11 package [18] was used for all phylogenetic analyses.

### 2.3. Biochemical Characteristics of FY-07

API 20 E system and API 50 CH system (bioMérieux, Lyon, France) were used to determine the biochemical characteristics of strain FY-07.

### 2.4. Comparative Genomics Analysis

The genomes sequences of 9 type species of *Kosakonia* were downloaded from Genebank and performed comparative genomics analysis with the FY-07 genome. ANI was calculated by fastANI software;while isDDH was conducted using GBDP by the TYPE (Strain) Genome Server [19]. One-hundred distance replicates were calculated each. The digital DDH values and confidence intervals were calculated using the recommended settings of the Genome-to-Genome Distance Calculator (GGDC 2.1) for GGDC formula 2 [20]. Four genomes that are more closely related to FY-07 and have high genome integrity were selected from *Kosakonia* genus for Genome synteny analyses. The genome synteny analyses were carried out by using Sibelia software and alignment of syntenic blocks was visualized in CGview [21,22]. 

### 2.5. Identification or Measurement of Plant Growth Promoting Traits

#### 2.5.1. IAA Production

Bacterial suspension of FY-07 was prepared as previously reported [12]. Then, 1 mL sterilized L- tryptophan stock solution (100 mg/mL) was added to the LB medium to make the final concentration reach 1 mg/mL. A 1% (*v*/*v*) inoculum of bacterial suspension was added to the LB medium with L- tryptophan and incubated at 30 °C in the dark for 10 days. Then, samples were centrifuged at 8000× *g* for 15 min, and 2 mL supernatant was collected in a new sterile tube and mixed with 2 mL Salkowski reagent. After 30 min of incubation in the dark, the absorbance of samples was measured immediately by using a spectrophotometer at the wavelength of 530 nm. Samples without L- tryptophan added were employed as the negative control. Three replicates were analyzed for each group of samples. IAA standard solutions (Aladdin, Shanghai, China) with concentrations of 0, 1, 3, 5, 10, 20, 50 and 100 μg/mL were used to draw the standard curve to calculate the IAA production of samples [23,24].

#### 2.5.2. Siderophore Production

Modified CAS Agar Medium Kit (Coolaber, Beijing, China) with chrome azurol S (CAS) and hexa decyl trimethyl ammonium bromide (HDTMA) as indicators [25] was used for siderophore production ability test. After the inoculation on the CAS medium at 30 °C for 5 days, the medium with yellowish-orange halo around the colonies was taken and photographed.

#### 2.5.3. Phosphate Solubilization

Pikovskaya medium (pH 7.2) composed of 1% glucose, 2.5% Ca_3_ (PO_4_)_2_, 0.05% (NH_4_)_2_ SO_4_, 0.02% NaCl, 0.01% MgSO_4_·7H_2_O, 0.02% KCl, 0.003% FeSO_4_·7H_2_O, 0.05% yeast extract, 0.003% MnSO_4_·4H_2_O, 2% agar with 0.24% bromophenol blue added was used for phosphorus solubilization ability test [26]. Next, 2.5 μL bacterial suspension of FY-07 was added on the Pikovskaya medium and incubated at 30 °C for 7 days. The plates were observed and photographed. The yellow area formed around the colony represented the dissolution of Ca_3_ (PO_4_)_2_.

To quantitatively estimate the ability of FY-07 to dissolve Ca_3_ (PO_4_)_2_, 1% bacterial suspension was inoculated into Pikovskaya medium without agar and cultured at 150 rpm for 10 days at 30 °C. The group without bacterial suspension addition was used as a control. Then, 4 mL culture medium was taken and centrifuged at 8000× *g* for 15 min. Subsequently, the content of soluble phosphorus in supernatant was determined by molybdenum antimony colorimetric method [27].

#### 2.5.4. Nitrogen Fixation

Jensen’s medium composed of 2% sucrose, 0.1% K_2_HPO_4_, 0.05% MgSO_4_, 0.05% NaCl, 0.01% FeSO_4_, 0.0005% Na_2_MoO_4_, 0.2% CaCO_3_ and 1.5% Agar was used for nitrogen fixation test. The ability of FY-07 to grow on Jensen’s medium represents its ability to fix nitrogen [28,29].

#### 2.5.5. Ammonia Production

FY-07 was cultured in LB medium as mentioned above for 72 h, and 2% (*v*/*v*) Nessler’s reagent was added to the culture supernatant. Subsequently, the absorbance of samples was measured at 425 nm. The uninoculated medium with Nessler’s reagent added was used as a control and ammonium chloride with concentrations from 0 to 300 μg/mL was used to draw the standard curve [30,31].

#### 2.5.6. ACC Deaminase Production

DF salt minimal medium composed of 0.4% KH_2_PO_4_, 0.6% Na_2_HPO_4_, 0.02% MgSO_4_·7H_2_0, 0.2% glucose, 0.2% sodium gluconate, 0.2% citric acid, 0.001% H_3_BO_3_, 0.0011% MnSO_4_·H_2_O, 0.0125% ZnSO_4_·7H_2_O, 0.0078% CuSO_4_·5H_2_O, 0.00001% MoO_3_ and 0.0001% FeSO_4_·7H_2_O was used for ACC deaminase production test [28,32]. Strains that grew significantly better on ADF (DF salt minimal medium with 3 mM ACC added) than DF salt minimal medium without ACC added were assumed to be able to use ACC as their sole source of nitrogen. 

### 2.6. Colonization of Rice Roots

The method was described by Zhang with modification [33]. For easier identification, a kanamycin resistant plasmid pBBR1-RFP constitutively expressing red fluorescent protein was transformed into FY-07. The overnight cultured strain was washed three times with physiological saline and adjusted its OD_600_ absorbance to 1 with physiological saline.

Rice seeds were soaked in 3% sodium hypochlorite solution for 5 min, rinsed with deionized water 3 times, soaked in 75% ethanol for 5 min, and rinsed with deionized water 5 times for surface disinfection. The disinfected seeds were placed in the bacterial suspension and inoculated at 180 rpm at 30 °C for 6 h. Seeds incubated in saline were used as controls. Seeds were then placed in Petri dishes with filter paper. After deionized water was added, seeds were grown in a lighted incubator until germination. The seeds were taken out of the incubator and moved in the plant hydroponic box. After 14 days of growth, deionized water was used to rinse the residual nutrient solution on the rice roots and the bacteria that are weakly adherent. After drying, the roots were put into a clean centrifuge tube and vortex with 1 mL sterile water to obtain bacterial suspension samples. Finally, samples were gradient diluted and plated on LB plates with and without kanamycin.

## 3. Results and Discussion

### 3.1. Phylogenetic Analysis

**Figure 1:**
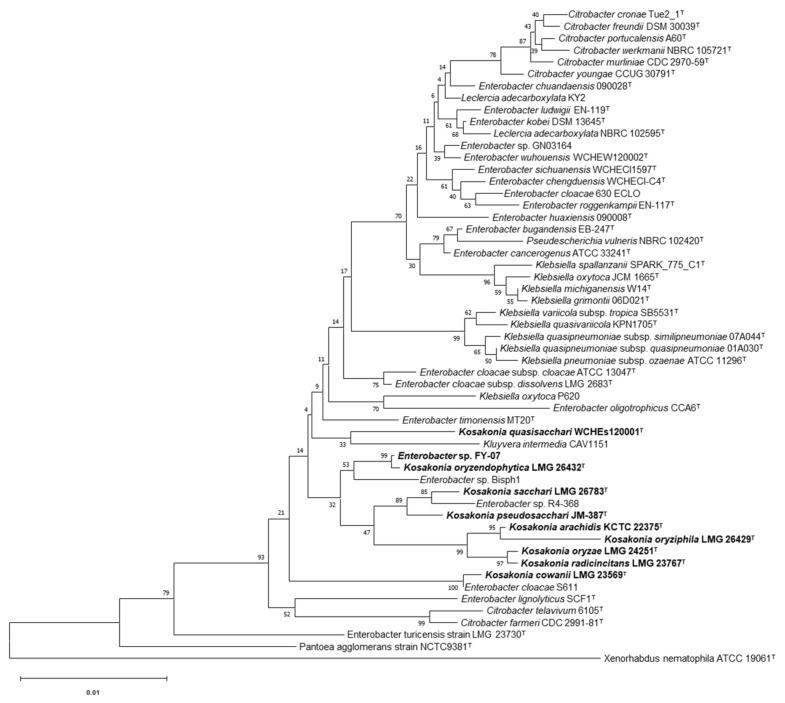
Neighbor-joining tree showing the 16S rRNA gene phylogenetic relationships of strain FY-07 and phylogenetically related reference strains. Bootstrap values based on 1000 resampling tests are shown at branch nodes. Bar, 1% substitution rate per site.

Consistent with the previous results [11], the 16S rRNA sequence alignment analysis showed that the strains with the highest similarity to FY-07 belonging to the *Enterobacteriaceae* and were close to *Enterobacter* (Figure 1). Phylogenetic analysis of the sequences of related strains found that FY-07 clearly formed a clade with the species of the genus *Kosakonia*, separated from other clades, and had the highest sequence similarity (99.93%) with *Kosakonia oryzendophytica* LMG 16432^T^ (Figure 1). These results indicated that FY-07 was closely related to the genus *Kosakonia*.

In addition, it could be seen from Figure 1 that *Enterobacter* sp. Bisph1 and *Enterobacter* sp. R4-368 formed a clade with FY-07 and other strains of the genus *Kosakonia*. This result suggested that *Enterobacter* sp. Bisph1 and *Enterobacter* sp. R4-368 should also probably be reclassified to the genus *Kosakonia*, and it is also indicated that there may be many bacteria in the genus *Enterobacter* that have not been correctly classified. More reclassification work should be done to aid research and applications of bacteria in *Enterobacteriaceae*.

To further determine the classification status of FY-07, a phylogenetic tree was reconstructed based on the connected partial *atpD*, *gyrB*, *infB* and *rpoB* gene sequences (Figure S1). The connected partial *atpD*, *gyrB*, *infB* and *rpoB* gene sequences analysis could clearly distinguish species in the family *Enterobacteriaceae*. This phylogenetic tree clearly showed that FY-07 forms a clade with strains of the genus *Kosakonia*, which is obviously separated from other genera of the *Enterobacteriaceae*. Among them, FY-07 and *Kosakonia* species *Kosakonia arachidis*, *Kosakonia cowanii*, *Kosakonia oryzae*, *Kosakonia oryziphila*, *Kosakonia oryzendophytica*, *Kosakonia pseudosacchari*, *Kosakonia radicincitans*, *Kosakonia sacchari* and *Kosakonia sacchari* showed 88.79–95.02% *atpD* gene sequence similarity, 86.39–98.65% *gyrB* gene sequence similarity, 87.48–99.19% *infB* gene sequence similarity, and 90.89–99.37% *rpoB* gene sequence similarity (Table S4). The strain FY-07 exhibited relatively low gene sequence similarities with *Enterobacter ludwigii* and *Enterobacter asburiae*, which were a species type of the *Enterobacter* genus (Table S4). These results confirmed the reliability of the MLSA method for identifying the taxonomic status of *Enterobacteriaceae*, and also strongly supported that the strain FY-07 should be transferred to the genus *Kosakonia*. In addition, consistent with the result of 16S rRNA sequence similarity, FY-07 and *Kosakonia oryzendophytica* showed the highest MLSA sequence similarity (98.07%), while the MLSA sequence similarities of FY-07 with other species in the genus were much lower (89.07–91.08%) (Table S5).

These phylogenetic analyses were sufficient to support that FY-07 should be transferred to the genus *Kosakonia*. However, the high 16S rRNA sequence similarity and MLSA sequence similarity to *Kosakonia oryzendophytica* made us wonder whether FY-07 is a new species in the genus *Kosakonia*, or it should be assigned to species *Kosakonia oryzendophytica*.

### 3.2. Comparative Genomics Analysis

**Figure 2:**
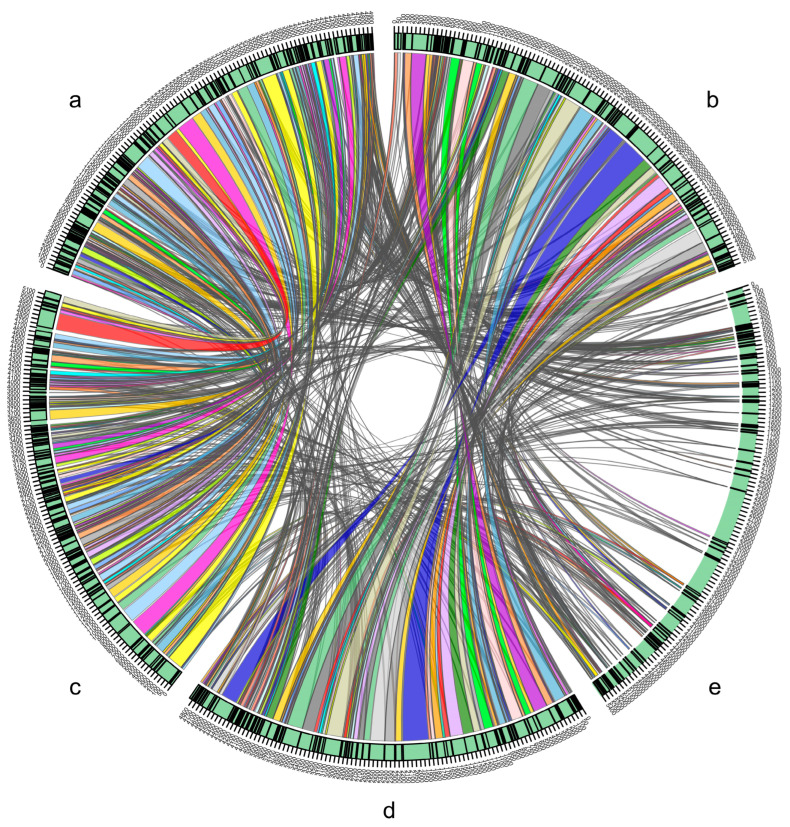
Genomic synteny shared between Kosakonia sacchari LMG 26783T (**a**), FY-07 (**b**), Kosakonia quasisacchari WCHEs120001T (**c**), Kosakonia oryzendophytica LMG 26432T (**d**) and Kosakonia oryzae LMG 24251T (**e**).

ANI values between FY-07 and other species except *Kosakonia oryzendophytica* in the genus *Kosakonia* were lower than the threshold (95%) [34], while the ANI value of FY-07 and *Kosakonia oryzendophytica* was 99.45%, which was much higher than the threshold (Table 1). GC content analysis showed that except for *Kosakonia arachidis* (1.08%) and *Kosakonia cowanii* (2.55%), the differences in GC content between FY-07 and other species in the *Kosakonia* genus were less than 1% (Table S6). Moreover, isDDH analysis showed FY-07 and *Kosakonia oryzendophytica* have the highest isDDH value (96.1%), which was far exceeding the threshold (70%), while the isDDH values of FY-07 and other species in the *Kosakonia* genus were much lower (25.3–26.5%) (Table 1). Genome synteny analyses indicated that FY-07 had the maximum synteny with *Kosakonia oryzendophytica* and the syntenic regions coverage was up to 98.00%, while syntenic regions coverage between FY-07 and *Kosakonia oryze*, *Kosakonia quasisacchari or Kosakonia sacchari* was only 37.52%, 20.30% and 19.49%, respectively (Figure 2). Clearly, the comparative genomics analyses indicated that FY-07 should be classified as a new strain of *Kosakonia oryzendophytica* rather than a new species in the genus *Kosakonia*.

In summary, on the basis of 16S rRNA sequence analysis, concatenated partial *atpD*, *gyrB*, *infB* and *rpoB* gene sequence analysis, ANI analysis, isDDH analysis and genome synteny analyses, *Enterobacter* sp. FY-07 should be reclassified as *Kosakonia oryzendophytica* comb. nov. and the type strain is REICA_082^T^ (= LMG 26432^T^ = NCCB 100390^T^). *Enterobacter* sp. FY-07 should be renamed *Kosakonia oryzendophytica* FY-07.

### 3.3. Biochemical Characteristics of FY-07

Using API strips can quickly complete the relevant biochemical reaction detection. Additionally, the biochemical characteristics of FY-07 were determined and the comparison with the strain types of all species in the genus *Kosakonia* was shown in Table 2. Briefly, except for very few differences, FY-07 has the same biochemical characteristics as *Kosakonia oryzendophytica*. Detailed results of the biochemical tests of FY-07 were given in the strain description.

### 3.4. Description of Kosakonia oryzendophytica FY-07

*Kosakonia oryzendophytica* (o.ryz.en.do.phy′ti.ca. L. fem. n. *oryza* rice; Gr. pref. *endo-* within; Gr. neut. n. *phyton* plant; L. masc. suff. *-icus* suffix used with the sense of pertaining to; N.L. fem. adj. *oryzendophytica* within rice plant, pertaining to the original isolation from rice tissues) [4].

Basonym: *Enterobacter* sp. FY-07 [11,12,13,14,15,35,36,37].

The description of *Kosakonia oryzendophytica* FY-07 is similar to that proposed for *Enterobacter oryzendophyticus* [38]. According to the experiment, it can be concluded that FY-07 has galactosidase, arginine dihydrolase and ornithine decarboxylase activities; the esculin hydrolysis, methyl red test, nitrate reduction, phosphate melting, indoleacetic acid and siderophore production positive reaction. It has no lysine decarboxylase, urease, tryptophan decarboxylase, gelatinase and oxidase activities. The production of hydrogen sulfide and indole in FY-07 is negative. Glucose, mannitol, sorbitol, sucrose, honey disaccharide, rhamnose, arabinose and amygdalin can be used for fermentation to produce acid, but inositol could not be used. The following substances could be used as the only carbon source for growth and development: glycerin, L-arabinose, D-ribose, D-xylose, D-galactose, D-glucose, D-fructose, D-mannose, α-methyl-D glucopyranoside, L-sorbose, L-rhamnose, D-mannitol, D-sorbitol, N-acetyl glucosamine, amygdalin, arbutin, esculine, salicin, D-cellobiose, maltose, lactose, sucrose, trehalose, gentian disaccharide, D-arabitol, gluconate, citric acid. However, erythritol, D- arabinose, L- xylose, adonitol, β-methyl-D xylopyranoside, dulcitol, inositol, D-melibiose, inulin, melezitose, raffinose, starch, glycogen, xylitol, D-pine disaccharide, D-Lyxose, D-tagatose, D-fucose, L-fucose, L-arabitol, 2-keto gluconate and 5-keto gluconate could not be used as the sole carbon source. The genomic DNA G + C content is 53.6%. 

### 3.5. Plant Growth-Promoting Ability of FY-07

The majority of the previous studies of FY-07 focused on the analyses of its unique BC production mechanism under anaerobic conditions [11,36], increasing the productivity [12,15] and expanding the application range of BC [13,14,35,37]. Herein, FY-07 was reclassified into the species *oryzendophytica* in the genus *Kosakonia* through a polyphasic approach. The model strain LMG 26432^T^ of the specie *Kosakonia oryzendophytica* was isolated from *Oryza sativa L.* [38]. Combined with the structural unity of BC and plant cellulose and the strong plant growth promoting ability of strains in the genus *Kosakonia*, we hypothesized that FY-07 has the potential to promote plant growth.

Previous reports revealed genetic basis for motility, competitiveness and plant growth-promoting capacities of *Kosakonia radicincitans* and its closely related bacteria through comparative genomics analyses [16]. The authors suggested that *Kosakonia radicincitans* shared the bulk of genes involved in plant growth-promotion with several strains including FY-07. According to this report, combined with genome analysis, we found that there are indeed many genes related to promoting plant growth in FY-07. The functions include nitrogen fixation, phosphorus solubilization, plant hormone synthesis, siderophore production, ACC deaminase production, betaine production, etc. The genes involved have been listed in Table 3. These large numbers of plant growth promoting related genes suggested that FY-07 may have a great potential for promoting plant growth. In particular, the genome of FY-07 contains fairly complete genes required for nitrogen fixation and siderophore production, which means that FY-07 is very likely to have strong nitrogen fixation and siderophore production capabilities. Therefore, the related traits of FY-07 were measured to further confirm its potential to promote plant growth.

**Figure 3:**
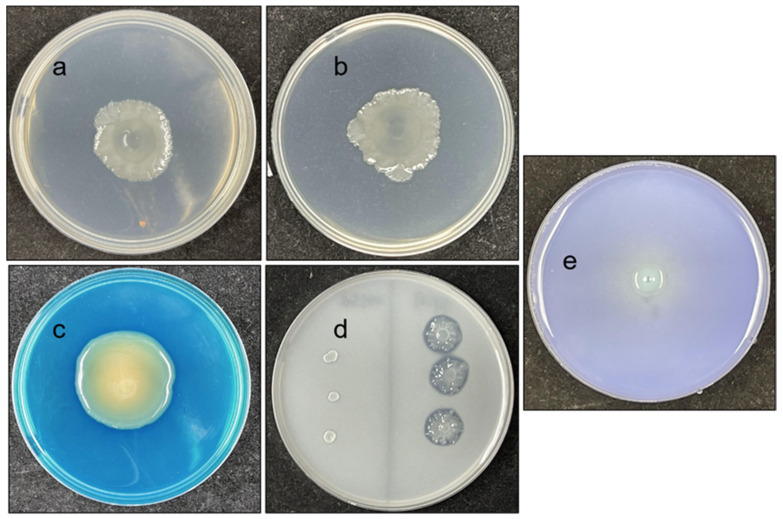
Identification of plant growth promoting traits of FY-07. (**a**), colony morphology of FY-07 on DF salt minimal medium without ACC added; (**b**), colony morphology of FY-07 on DF medium with ACC; (**c**), colony morphology of FY-07 on Modified CAS Agar Medium; (**d**), comparison of colony morphology between *Escherichia coli* MG1655 (left) and FY-07 (right) on Jensen’s medium; (**e**), colony morphology of FY-07 on Pikovskaya medium.

As shown in Figure 3, common significant plant growth-promoting traits were present in FY-07. Compared with other reported plant growth-promoting bacteria, FY-07 has a relatively high ability to dissolve insoluble phosphate (282 ± 9.9 μg/mL), produce ammonium (32 ± 0.4 mM) and produce IAA (19 ± 0.7μg/mL) (Figure 3e) [38,39]. The better growth on medium added with ACC indicated that FY-07 could produce ACC deaminase (Figure 3a,b). The strong growth on Jensen’s medium and the huge yellowish orange halo on CAS medium also indicated that FY-07 has a strong nitrogen fixation ability and siderophore production ability (Figure 3c,d). These results suggested that, as a member of the genus *Kosakonia*, FY-07 indeed has a strong plant growth-promoting potential like most other members of the genus.

**Figure 4:**
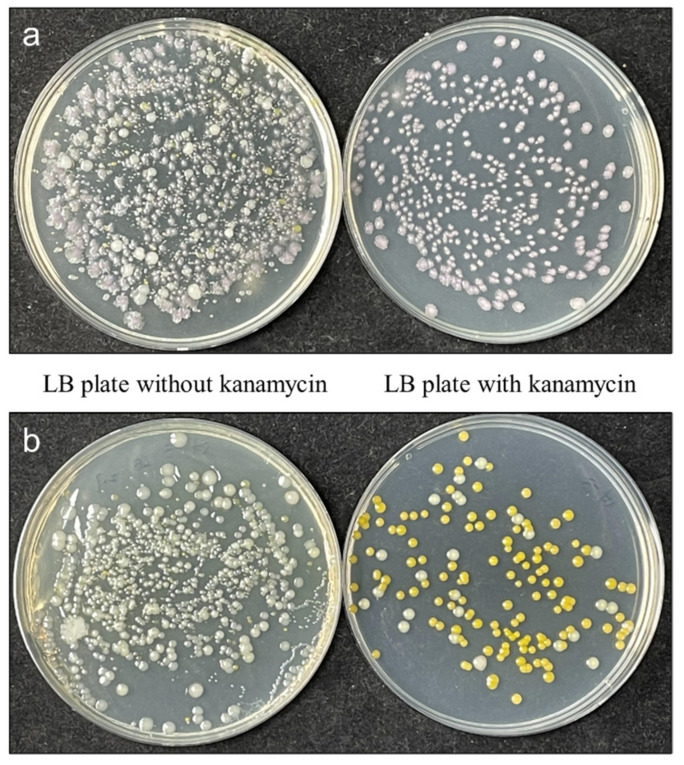
Number and morphology of colonies from rice roots with (**a**) and without (**b**) FY-07 treatment grown on LB plates with (right) and without (left) kanamycin.

As FY-07 was reclassified to *Kosakonia oryzendophytica*, Rice (*Oryzasativa* spp. *Japonica*, L.) was selected as the study object to further examine the interaction of FY-07 with plants. As shown in Figure 4, there were a large number (about 0.8 × 10^6^) of microorganisms in different forms in the roots of rice in the control group, in which two kinds of bacteria (shown as yellow and white colonies) could grow in the presence of kanamycin. After FY-07 treatment, there were also a large number (about 1 × 10^6^) of microorganisms in rice roots, of which about 46% are FY-07 (shown as red colonies), and only FY-07 could grow in the presence of kanamycin. These results indicated that there is a complex flora in the rice roots themselves, and FY-07 can indeed colonize rice seeds after incubation and enrich in the roots as the rice grows. 

A high abundance of certain genera in plant microbiomes protects host plants from disease. These bacteria pass through generations in seeds. [40]. *Kosakonia radicincitans* enhances a plant’s resistance against phloem-feeding and chewing insects in *Arabidopsis thaliana* [41]. These reports indicated that the FY-07-dominated flora formed in the roots will undoubtedly have a significant impact on the growth of plants. This flora may also help plants resist disease and environmental stress. However, since nutrition was sufficient in the early stage of growth, there was no significant difference in the growth status of rice between the experimental and control groups at 14 days. 

Furthermore, although abundant in the atmosphere, plants cannot use nitrogen directly for growth, so microbial nitrogen fixation is particularly important [42]. Unlike the nodulating rhizobial associations, many nitrogen-fixing bacterial groups including *Enterobacter* and *Gluconacetobacter* may have the ability to establish associative and endophytic associations with plants [43]. However, assignment as an association or endophytic colonization is not always well defined, because bacterial niche and numbers can be dynamically controlled during plant-bacteria interactions in response to plant and environmental signals [44]. In short, *Kosakonia*, which is closely related to *Enterobacter*, is likely to be associative nitrogen-fixing bacteria or endophytic nitrogen-fixing bacteria. Matthias et al. showed that *Kosakonia radicincitans* DSM 16656^T^ was initially attracted to root hairs when interacting with tomatoes, and entered the plant through cracks around the newly emerging lateral roots, and eventually colonized the interior of individual root parenchyma cells [16]. Such a life cycle in response to plant and environmental signals is very consistent with the characteristics of associative nitrogen-fixing bacteria or endophytic nitrogen-fixing bacteria [44]. We believe that FY-07 is also an associative nitrogen-fixing bacteria or endophytic nitrogen-fixing bacteria. Strong motility can help FY-07 adapt to different environments and respond to signals of plant host in a timely manner, and the production of BC after reaching the vicinity of the host will help colonize and protect itself. Subsequently, strong abilities of FY-07 such as nitrogen fixation, phosphate solubilization, hormone production, etc., can promote plant growth, while FY-07 can obtain carbon sources from the plant host to maintain their own growth and metabolism. Afterwards, under the influence of environmental signals, quorum sensing signals or host signals, FY-07 will leave the host and re-enter the environment to find new hosts. The above inferred life cycle based on the characteristics of associative nitrogen-fixing bacteria can well explain the unique characteristics of BC produced by FY-07 [15]. More experiments and related data will further reveal the relevant principles and mechanisms. 

In addition, as model strains for BC production, many strains in *Gluconacetobacter* are also isolated from plants, and we believe that further research on the interaction mechanism between FY-07 and plants is of great significance not only for *Kosakonia* genus but also for *Gluconacetobacter* genus. Additionally, our research has important implications for the future application of *Kosakonia* genus in agriculture.

## 4. Conclusions

In this study, phylogeny of *Enterobacter* sp. FY-07 based on 16S rRNA genes, MLSA and complete genomes was investigated by using a polyphasic approach. Results revealed that *Enterobacter* sp. FY-07 was suggested to be reclassified as *Kosakonia oryzendophytica* FY-07. Several plant growth-promoting traits of FY-07 were identified and the colonization of FY-07 in rice roots was also confirmed. It was proved that FY-07 has relatively high ability to dissolve insoluble phosphate, produce ammonium and IAA. Additionally, FY-07 has a strong nitrogen fixation ability and siderophore production ability. All results indicated that FY-07 has the potential to promote plant growth and hinted that the interaction between plants and FY-07 is related to its unique life cycle.

## Figures and Tables

**Table 1:** Pairwise comparisons of isDDH and ANI values of FY-07 and type strains of the genus Kosakonia.

= ANI ≥ 95% or isDDH ≥ 70%	*Enterobacter* sp. FY-07	*Kosakonia oryzendophytica* LMG 26432^T^	*Kosakonia sacchari* LMG 26783^T^	*Kosakonia pseudosacchari* JM-387^T^	*Kosakonia oryzae* LMG 24251^T^	*Kosakonia quasisacchari* WCHEs 120001^T^	*Kosakonia radicincitans* LMG 23767^T^	*Kosakonia oryziphila* LMG 26429^T^	*Kosakonia arachidis* KCTC 22375^T^
*Enterobacter* sp. FY-07		96.10	26.50	26.40	26.40	26.10	26.50	25.60	25.30
*Kosakonia* *oryzendophytica* LMG 26432^T^	99.45		26.20	26.30	26.20	26.10	26.20	25.60	25.30
*Kosakonia sacchari* LMG 26783^T^	84.13	84.21		59.10	28.00	50.90	27.80	26.70	26.60
*Kosakonia pseudosacchari* JM-387^T^	84.07	84.08	94.94		27.80	48.60	27.70	27.00	26.50
*Kosakonia oryzae* LMG 24251^T^	83.89	83.89	84.95	84.74		27.40	66.40	53.10	51.30
*Kosakonia quasisacchari* WCHEs 120001^T^	83.83	83.90	93.29	92.71	84.45		27.40	26.60	26.40
*Kosakonia* *radicincitans* LMG 23767^T^	83.78	83.78	84.66	84.52	95.85	84.36		54.30	52.40
*Kosakonia oryziphila* LMG 26429^T^	83.08	83.15	83.99	84.13	92.92	83.80	93.04		54.80
*Kosakonia arachidis* KCTC 22375^T^	82.78	82.99	83.86	83.80	92.62	83.69	92.80	93.36	
*Kosakonia cowanii* LMG 23569^T^	82.37	82.48	83.66	83.71	83.17	83.27	82.98	82.39	82.10

The upper triangle (blue portion) displays isDDH values (%), and the lower triangle (yellow portion) displays ANI values (%). Boxes with isDDH ≥ 70% or ANI ≥ 95% are colored red.

**Table 2:** Phenotypic features of strain FY-07 and strain types of *Kosakonia* species [4,5,6,7]: 1, FY-07; 2, *Kosakonia oryzendophytica*; 3, *Kosakonia sacchari*; 4, *Kosakonia pseudosacchari*; 5, *Kosakonia oryzae*; 6, *Kosakonia quasisacchari*; 7, Kosakonia radicincitans; 8, *Kosakonia oryziphila*; 9, *Kosakonia arachidis*; 10, *Kosakonia cowanii*. +, positive; −, negative.

Characteristic	1	2	3	4	5	6	7	8	9	10
β-Galactosidase	+	+	+	+	+	+	+	+	+	+
Arginine dihydrolase	+	+	−	+	+	+	+	+	+	+
Lysine decarboxylase	−	−	−	−	−	−	−	−	−	−
Ornithine decarboxylase	+	+	+	−	+	−	−	−	+	−
Citrate utilization	+	+	+	+	+	+	+	+	+	+
H_2_S production	−	−	−	−	−	−	−	−	−	−
Urea hydrolysis	−	−	−	−	−	−	−	−	−	−
Indole production	−	−	−	−	−	−	−	−	−	−
Voges-Proskauer reaction	+	+	+	+	+	+	+	+	+	+
Gelatinase	−	−	−	−	−	−	−	−	−	−
d-Glucose	+	+	+	+	+	+	+	+	+	+
d-Mannitol	+	+	+	+	+	+	+	+	+	+
Inositol	−	+	−	−	−	−	−	+	+	−
d-Sorbitol	+	+	+	+	+	+	+	+	+	+
Sucrose	+	+	+	+	+	+	+	+	+	+
Melibiose	+	+	+	−	+	−	−	−	−	+

**Table 3:** Genes of FY-07 involved in plant growth-promotion.

Traits	Genes
Nitrogen fixation and metabolism	*amtB*, *narB*, *narG*, *narH*, *narI*, *narJ*, *nark*, *narL*, *narX*, *nasD*, *nifA*, *nifB*, *nifD*, *nifE*, *nifF*, *nifH*, *nifI*, *nifJ*, *nifK*, *nifL*, *nifK*, *nifL*, *nifM*, nifN, *nifQ*, *nifS*, *nifT*, *nifU*, *nifV*, *nifW*, *nifX*, *nifZ*, *nirB*, *nirD*, *ntrA*, *ntrB*, *ntrC*
Phosphate solubilization	*phoA*, *phoB*, *phoC*, *phoE*, *phoH*, *phoR*, *phoU*, *pqqE*-like, *pstA*, *pstB*, *pstC*, *pstS*
Plant hormone synthesis	*ipdC*, *gabD*, *puuE*
Siderophore production	*acrB*, *efeB*, *efeO*, *efeU*, *entA*, *entB*, *entC*, *entE*, *entF*, *entH*, *entS*, *exbB*, *exbD*, *feoA*, *feoB*, *feoC*, *fepA*, *fepB*, *fepC*, *fepD*, *fepE*, *fepG*, *fes*, *fhuB*, *fhuC*, *fhuE*, *fhuF*, *fptA*, *mbtH*-like, *mdtA*, *mdtB*, *mdtC*, *mexA*, *mexB*, *tonB*, *yusV*
ACC deaminase production	*dcyD*
Betaine production	*opuA*, *opuB*, *opuC*, *osmY*, *proV*, *proW*, *proX*
Acetoin and butanodiol sythesis	*als*, *budA*, *budB*, *budC*, *poxB*

## Data Availability

The GenBank accession numbers for the *atpD*, *gyrB*, *infB* and *rpoB* gene sequences of the strain FY-07 are OM049817.1–OM049820.1. The GenBank accession number for the whole-genome sequences of the strain FY-07 is CP012487.1. Six supplementary tables and one supplementary figure are available with the online version of this paper.

## Supplementary Materials

The following supporting information can be downloaded at: https://www.mdpi.com/article/10.3390/microorganisms10030575/s1, Figure S1: Neighbour-joining tree showing the phylogenetic relationships of strain FY-07 and phylogenetically related reference strains based on concatenated partial *atpD*, *gyrB*, *infB* and *rpoB* gene sequences. Bootstrap values based on 1000 resamplings are shown at branch nodes. Bar, 2% substitution rate per site; Table S1: The GenBank/EMBL/DDBJ accession numbers for 16S rRNA gene sequences used in this study; Table S2: The GenBank/EMBL/DDBJ accession numbers for *atpD*, *gyrB*, *infB* and *rpoB* gene sequences used in this study; Table S3: The GenBank/EMBL/DDBJ accession numbers for genome sequences used in this study; Table S4: *atpD*, *gyrB* and *infB*, *rpoB* gene sequence similarities between strain FY-07 and type strains of *Kosakonia* and other phylogenetically related species; Table S5: Pairwise similarities of concatenated partial *atpD*, *gyrB*, *infB* and *rpoB* gene sequences for type strains of the genus *Kosakonia*; Table S6: The difference of G + C content between FY-07 and type strains of the genus *Kosakonia*.
