# Supplementary material for: Reclassification of Enterobacter sp. FY-07 as Kosakonia oryzendophytica FY-07 and Its Potential to Promote Plant Growth
Source: Microorganisms. 2022 Mar 6;10(3):575. doi: 10.3390/microorganisms10030575 (PMC8951479; doi:10.3390/microorganisms10030575)
Supplement: Supplementary file 1 [file microorganisms-10-00575-s001.zip › microorganisms-1578746-supplementary.pdf]

Reclassification of the strain *Enterobacter* sp. FY-07 as *Kosakonia oryzendophytica* FY-07 and its Potential to Promote Plants Growth

Ge Gao[a, †], Yan Zhang[a, †], Shaofang Niu[a], Yu Chen[a], Shaojing Wang[a], Nusratgul Anwar[a], Shuai Chen[a], Guoqiang Li[a, b]*, Ting Ma[a, b]*

Affiliations

[a] Key Laboratory of Molecular Microbiology and Technology, Ministry of Education, College of Life Sciences, Nankai University, Tianjin 300071, China

[b] Tianjin Engineering Technology Center of Green Manufacturing Biobased Materials, Tianjin 300071, China

†These authors contributed equally to this paper.

***Corresponding author**

Guoqiang Li: gqli@nankai.edu.cn

Ting Ma: tingma@nankai.edu.cn; Phone: 86-22-23508870; Fax: 86-22-23508870.

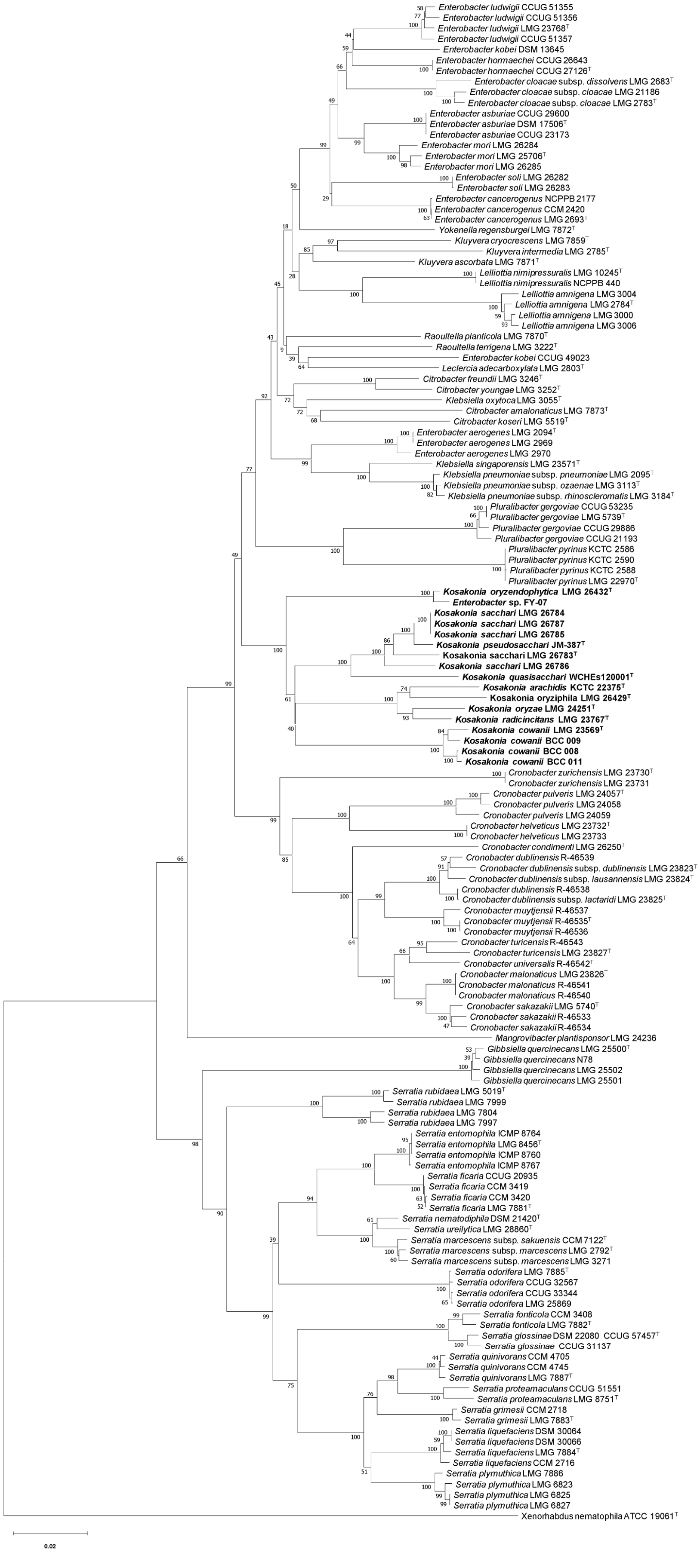

Enterobacter ludwigii CCUG 51355
Enterobacter ludwigii CCUG 51356
Enterobacter ludwigii LMG 23768^{T}
Enterobacter ludwigii CCUG 51357
Enterobacter kobei DSM 13645
Enterobacter hormaechei CCUG 26643
Enterobacter hormaechei CCUG 27126^{T}
Enterobacter cloacae subsp. dissolvens LMG 2683^{T}
Enterobacter cloacae subsp. cloacae LMG 21186
Enterobacter cloacae subsp. cloacae LMG 2783^{T}
Enterobacter asburiae CCUG 29600
Enterobacter asburiae DSM 17506^{T}
Enterobacter asburiae CCUG 23173
Enterobacter mori LMG 26284
Enterobacter mori LMG 25706^{T}
Enterobacter mori LMG 26285
Enterobacter soli LMG 26282
Enterobacter soli LMG 26283
Enterobacter cancerogenus NCPPB 2177
Enterobacter cancerogenus CCM 2420
Enterobacter cancerogenus LMG 2693^{T}
Yokenella regensburgei LMG 7872^{T}
Kluyvera cryocrescens LMG 7859^{T}
Kluyvera intermedia LMG 2785^{T}
Kluyvera ascorbata LMG 7871^{T}
Lelliottia nimipressuralis LMG 10245^{T}
Lelliottia nimipressuralis NCPPB 440
Lelliottia amnigena LMG 3004
Lelliottia amnigena LMG 2784^{T}
Lelliottia amnigena LMG 3000
Lelliottia amnigena LMG 3006
Raoultella planticola LMG 7870^{T}
Raoultella terrigena LMG 3222^{T}
Enterobacter kobei CCUG 49023
Leclercia adecarboxylata LMG 2803^{T}
Citrobacter freundii LMG 3246^{T}
Citrobacter youngae LMG 3252^{T}
Klebsiella oxytoca LMG 3055^{T}
Citrobacter amalonaticus LMG 7873^{T}
Citrobacter koseri LMG 5519^{T}
Enterobacter aerogenes LMG 2094^{T}
Enterobacter aerogenes LMG 2969
Enterobacter aerogenes LMG 2970
Klebsiella singaporensis LMG 23571^{T}
Klebsiella pneumoniae subsp. pneumoniae LMG 2095^{T}
Klebsiella pneumoniae subsp. ozaenae LMG 3113^{T}
Klebsiella pneumoniae subsp. rhinoscleromatis LMG 3184^{T}
Pluralibacter gergoviae CCUG 53235
Pluralibacter gergoviae LMG 5739^{T}
Pluralibacter gergoviae CCUG 29886
Pluralibacter gergoviae CCUG 21193
Pluralibacter pyrinus KCTC 2586
Pluralibacter pyrinus KCTC 2590
Pluralibacter pyrinus KCTC 2588
Pluralibacter pyrinus LMG 22970^{T}
Kosakonia oryzendophytica LMG 26432^{T}
Enterobacter sp. FY-07
Kosakonia sacchari LMG 26784
Kosakonia sacchari LMG 26787
Kosakonia sacchari LMG 26785
Kosakonia pseudosacchari JM-387^{T}
Kosakonia sacchari LMG 26783^{T}
Kosakonia sacchari LMG 26786
Kosakonia quasisacchari WCHEs120001^{T}
Kosakonia arachidis KCTC 22375^{T}
Kosakonia oryziphila LMG 26429^{T}
Kosakonia oryzae LMG 24251^{T}
Kosakonia radicincitans LMG 23767^{T}
Kosakonia cowanii LMG 23569^{T}
Kosakonia cowanii BCC 009
Kosakonia cowanii BCC 008
Kosakonia cowanii BCC 011
Cronobacter zurichensis LMG 23730^{T}
Cronobacter zurichensis LMG 23731
Cronobacter pulveris LMG 24057^{T}
Cronobacter pulveris LMG 24058
Cronobacter pulveris LMG 24059
Cronobacter helveticus LMG 23732^{T}
Cronobacter helveticus LMG 23733
Cronobacter condimenti LMG 26250^{T}
Cronobacter dublinensis R-46539
Cronobacter dublinensis subsp. dublinensis LMG 23823^{T}
Cronobacter dublinensis subsp. lausannensis LMG 23824^{T}
Cronobacter dublinensis R-46538
Cronobacter dublinensis subsp. lactaridi LMG 23825^{T}
Cronobacter muytjensii R-46537
Cronobacter muytjensii R-46535^{T}
Cronobacter muytjensii R-46536
Cronobacter turicensis R-46543
Cronobacter turicensis LMG 23827^{T}
Cronobacter universalis R-46542^{T}
Cronobacter malonaticus LMG 23826^{T}
Cronobacter malonaticus R-46541
Cronobacter malonaticus R-46540
Cronobacter sakazakii LMG 5740^{T}
Cronobacter sakazakii R-46533
Cronobacter sakazakii R-46534
Mangrovibacter plantisponsor LMG 24236
Gibbsiella quercinecans LMG 25500^{T}
Gibbsiella quercinecans N78
Gibbsiella quercinecans LMG 25502
Gibbsiella quercinecans LMG 25501
Serratia rubidaea LMG 5019^{T}
Serratia rubidaea LMG 7999
Serratia rubidaea LMG 7804
Serratia rubidaea LMG 7997
Serratia entomophila ICMP 8764
Serratia entomophila LMG 8456^{T}
Serratia entomophila ICMP 8760
Serratia entomophila ICMP 8767
Serratia ficaria CCUG 20935
Serratia ficaria CCM 3419
Serratia ficaria CCM 3420
Serratia ficaria LMG 7881^{T}
Serratia nematodiphila DSM 21420^{T}
Serratia ureilytica LMG 28860^{T}
Serratia marcescens subsp. sakuensis CCM 7122^{T}
Serratia marcescens subsp. marcescens LMG 2792^{T}
Serratia marcescens subsp. marcescens LMG 3271
Serratia odorifera LMG 7885^{T}
Serratia odorifera CCUG 32567
Serratia odorifera CCUG 33344
Serratia odorifera LMG 25869
Serratia fonticola CCM 3408
Serratia fonticola LMG 7882^{T}
Serratia glossinae DSM 22080 CCUG 57457^{T}
Serratia glossinae CCUG 31137
Serratia quinivorans CCM 4705
Serratia quinivorans CCM 4745
Serratia quinivorans LMG 7887^{T}
Serratia proteamaculans CCUG 51551
Serratia proteamaculans LMG 8751^{T}
Serratia grimesii CCM 2718
Serratia grimesii LMG 7883^{T}
Serratia liquefaciens DSM 30064
Serratia liquefaciens DSM 30066
Serratia liquefaciens LMG 7884^{T}
Serratia liquefaciens CCM 2716
Serratia plymuthica LMG 7886
Serratia plymuthica LMG 6823
Serratia plymuthica LMG 6825
Serratia plymuthica LMG 6827
Xenorhabdus nematophila ATCC 19061^{T}
0.02

Figure S1. Neighbour-joining tree showing the phylogenetic relationships of strain FY-07 and phylogenetically related reference strains based on concatenated partial *atpD*, *gyrB*, *infB* and *rpoB* gene sequences. Bootstrap values based on 1000 resamplings are shown at branch nodes. Bar, 2% substitution rate per site.

Table S1. The GenBank/EMBL/DDBJ accession numbers for 16S rRNA gene sequences used in this study.

**Strains**	**Accession Number**
Citrobacter cronae Tue2_1^T	MN548424.1
Citrobacter farmeri CDC 2991-81^T	AF025371.1
Citrobacter freundii DSM 30039^T	AJ233408.1
Citrobacter murliniae CDC 2970-59^T	AF025369.1
Citrobacter portucalensis A60^T	MVFY01000035.1
Citrobacter telavivum 6105^T	MN603664.1
Citrobacter werkmanii NBRC 105721^T	BBMW01000025.1
Citrobacter youngae CCUG 30791^T	RPOI01000045.1
Cronobacter zurichensis LMG 23730T	HQ992947.1
Enterobacter bugandensis EB-247^T	FYBI01000003.1
Enterobacter cancerogenus ATCC 33241^T	FYBA01000020.1
Enterobacter chengduensis WCHECl-C4^T	KY979142.1
Enterobacter chuandaensis 090028^T	MK049966.1
Enterobacter cloacae 630 ECLO	JUZJ01000090.1
Enterobacter cloacae S611	AXOM01000004.1
Enterobacter cloacae subsp. *cloacae* ATCC 13047^T	CP001918.1
Enterobacter cloacae subsp. *dissolvens* LMG 2683^T	Z96079.1
Enterobacter huaxiensis 090008^T	MK049964.1
Enterobacter kobei DSM 13645^T	CP017181.1
Enterobacter lignolyticus SCF1^T	CP002272.1
Enterobacter ludwigii EN-119^T	JTLO01000001.1
Enterobacter oligotrophicus CCA6^T	LC368255.1
Enterobacter roggenkampii EN-117^T	CP017184.1
Enterobacter sichuanensis WCHECl1597^T	POVL01000141.1
Enterobacter sp. Bisph1	JXAG01000010.1
Enterobacter sp. R4-368	CP005991.1
Enterobacter sp.GN03164	LECZ01000016.1
Enterobacter timonensis MT20^T	FCOP01000010.1
Enterobacter wuhouensis WCHEW120002^T	SJOO01000031.1
Klebsiella grimontii 06D021^T	FZTC01000044.1
Klebsiella michiganensis W14^T	JQ070300.1
Klebsiella oxytoca JCM 1665^T	AB004754.1
Klebsiella oxytoca P620	CP046115.1
Klebsiella pneumoniae subsp. *ozaenae* ATCC 11296^T	Y17654.1
Klebsiella quasipneumoniae subsp. *quasipneumoniae* 01A030^T	HG933296.1
Klebsiella quasipneumoniae subsp. *similipneumoniae* 07A044^T	CBZR010000040.1

Klebsiella quasivariicola KPN1705^T	CP022823.1
Klebsiella spallanzanii SPARK_775_C1^T	MN091365.1
Klebsiella variicola subsp. *tropica* SB5531^T	CAAHGN010000012.1
Kluyvera intermedia CAV1151	CP011602.1
Kosakonia arachidis KCTC 22375^T	NR_116403.1
Kosakonia cowanii LMG 23569^T	NR_025566.1
Kosakonia oryzae LMG 24251^T	CP014007.1
Kosakonia oryzendophytica LMG 26432^T	JF795011.1
Kosakonia oryziphila LMG 26429^T	JF795013.1
Kosakonia pseudosacchari JM-387^T	FXWP01000029.1
Kosakonia quasisacchari WCHEs120001^T	MK567956.1
Kosakonia radicincitans LMG 23767^T	CP018016.1
Kosakonia sacchari LMG 26783^T	CP007215.1
Leclercia adecarboxylata KY2	JWJV01000013.1
Leclercia adecarboxylata NBRC 102595^T	BCNP01000062.1
Pantoea agglomerans NCTC 9381T	AJ251466.1
Pseudescherichia vulneris NBRC 102420^T	BBMZ01000044.1
Xenorhabdus nematophila ATCC 19061T	AY278674.1

Table S2. The GenBank/EMBL/DDBJ accession numbers for *atpD*, *gyrB*, *infB* and *rpoB* gene sequences used in this study.

**Strains**	***atpD***	***gyrB***	***infB***	***rpoB***
Citrobacter amalonaticus strain LMG 7873^T	JX424962.1	JX425091.1	JX425221.1	JX425347.1
Citrobacter freundii strain LMG 3246^T	JX424960.1	JX425089.1	JX425219.1	JX425345.1
Citrobacter koseri strain LMG 5519^T	JX424961.1	JX425090.1	JX425220.1	JX425346.1
Citrobacter youngae strain LMG 3252^T	JX424963.2	JX425092.1	JX425222.1	JX425348.1
Cronobacter condimenti strain LMG 26250^T	JX424906.1	JX425036.1	JX425165.1	JX425292.1
Cronobacter dublinensis strain R-46538	JX424898.1	JX425028.1	JX425157.1	JX425284.1
Cronobacter dublinensis strain R-46539	JX424899.1	JX425029.1	JX425158.1	JX425285.1
Cronobacter dublinensis subsp. *dublinensis* LMG 23823^T	JX424895.1	JX425025.1	JX425154.1	JX425281.1
Cronobacter dublinensis subsp. *lactaridi* LMG 23825^T	JX424897.1	JX425027.1	JX425156.1	JX425283.1
Cronobacter dublinensis subsp. *lausannensis* LMG 23824^T	JX424896.1	JX425026.1	JX425155.1	JX425282.1
Cronobacter helveticus strain LMG 23732^T	JX424907.1	JX425037.1	JX425166.1	JX425293.1
Cronobacter helveticus strain LMG 23733	JX424908.1	JX425038.1	JX425167.1	JX425294.1
Cronobacter malonaticus LMG 23826^T	JX424900.1	JX425030.1	JX425159.1	JX425286.1
Cronobacter malonaticus strain R-46540	JX424901.1	JX425031.1	JX425160.1	JX425287.1
Cronobacter malonaticus strain R-46541	JX424902.1	JX425032.1	JX425161.1	JX425288.1
Cronobacter muytjensii strain R-46535^T	JX424892.1	JX425022.1	JX425151.1	JX425278.1
Cronobacter muytjensii strain R-46536	JX424893.1	JX425023.1	JX425152.1	JX425279.1
Cronobacter muytjensii strain R-46537	JX424894.1	JX425024.1	JX425153.1	JX425280.1
Cronobacter pulveris strain LMG 24057^T	JX424909.1	JX425039.1	JX425168.1	JX425295.1

Cronobacter pulveris strain LMG 24058[T]	JX424910.1	JX425040.1	JX425169.1	JX425296.1
Cronobacter pulveris strain LMG 24059[T]	JX424911.1	JX425041.1	JX425170.1	JX425297.1
Cronobacter sakazakii strain LMG 5740[T]	JX424889.1	JX425019.1	JX425148.1	JX425275.1
Cronobacter sakazakii strain R-46533	JX424890.1	JX425020.1	JX425149.1	JX425276.1
Cronobacter sakazakii strain R-46534	JX424891.1	JX425021.1	JX425150.1	JX425277.1
Cronobacter turicensis strain R-46543	JX424905.1	JX425035.1	JX425164.1	JX425291.1
Cronobacter turicensis z3032 strain LMG 23827[T]	JX424904.1	JX425034.1	JX425163.1	JX425290.1
Cronobacter universalis strain R-46542[T]	JX424903.1	JX425033.1	JX425162.1	JX425289.1
Cronobacter zurichensis strain LMG 23730[T]	JX424912.1	JX425042.1	JX425171.1	JX425298.1
Cronobacter zurichensis strain LMG 23731	JX424913.1	JX425043.1	JX425172.1	JX425299.1
Enterobacter aerogenes strain LMG 2094[T]	JX424969.1	JX425098.1	JX425228.1	JX425354.1
Enterobacter aerogenes strain LMG 2969	JX424970.1	JX425099.1	JX425229.1	JX425355.1
Enterobacter aerogenes strain LMG 2970	JX424971.1	JX425100.1	JX425230.1	JX425356.1
Enterobacter asburiae strain CCUG 23173	JX424860.1	JX424990.1	JX425119.1	JX425249.1
Enterobacter asburiae strain CCUG 29600	JX424861.1	JX424991.1	JX425120.1	JX425250.1
Enterobacter asburiae strain DSM 17506[T]	JX424859.1	JX424989.1	JX425118.1	JX425248.1
Enterobacter cancerogenus strain CCM 2420	JX424851.1	JX424981.1	JX425110.1	JX425240.1
Enterobacter cancerogenus strain LMG 2693[T]	JX424850.1	JX424980.1	JX425109.1	JX425239.1
Enterobacter cancerogenus strain NCPPB 2177	JX424852.1	JX424982.1	JX425111.1	JX425241.1
Enterobacter cloacae subsp. *cloacae* strain LMG 21186	JX424848.1	JX424978.1	JX425107.1	JX425237.1
Enterobacter cloacae subsp. *cloacae* strain LMG 2783[T]	JX424847.1	JX424977.1	JX425106.1	JX425236.1
Enterobacter cloacae subsp. *dissolvens* strain LMG 2683[T]	JX424849.1	JX424979.1	JX425108.1	JX425238.1
Enterobacter hormaechei strain CCUG 26643	JX424854.1	JX424984.1	JX425113.1	JX425243.1
Enterobacter hormaechei strain CCUG 27126[T]	JX424853.1	JX424983.1	JX425112.1	JX425242.1
Enterobacter kobei strain CCUG 49023	JX494748.1	JX494750.1	JX494751.1	JX494753.1
Enterobacter kobei strain DSM 13645	JX494747.1	JX494749.1	JX494752.1	JX494754.1
Enterobacter ludwigii strain CCUG 51355	JX424856.1	JX424986.1	JX425115.1	JX425245.1
Enterobacter ludwigii strain CCUG 51356	JX424857.1	JX424987.1	JX425116.1	JX425246.1
Enterobacter ludwigii strain CCUG 51357	JX424858.1	JX424988.1	JX425117.1	JX425247.1
Enterobacter ludwigii strain LMG 23768[T]	JX424855.1	JX424985.1	JX425114.1	JX425244.1
Enterobacter mori LMG 25706[T]	JX424862.1	JX424992.1	JX425121.1	JX425251.1
Enterobacter mori strain LMG 26284	JX424864.1	JX424994.1	JX425123.1	JX425253.1
Enterobacter mori strain LMG 26285	JX424863.1	JX424993.1	JX425122.1	JX425252.1
Enterobacter soli strain LMG 26282	JX424865.1	JX424995.1	JX425124.1	JX425254.1
Enterobacter soli strain LMG 26283	JX424866.1	JX424996.1	JX425125.1	JX425255.1
Enterobacter sp. strain FY-07	OM049817.1	OM049818.1	OM049819.1	OM049820.1
Gibbsiella quercinecans strain LMG 25500[T]	JX424954.1	JX425084.1	JX425213.1	JX425340.1
Gibbsiella quercinecans strain LMG 25501	JX424955.1	JX425085.1	JX425214.1	JX425341.1
Gibbsiella quercinecans strain LMG 25502	JX424956.1	JX425086.1	JX425215.1	JX425342.1
Gibbsiella quercinecans strain N78[T]	JX424957.1	GU562335.1	JX425216.1	GU562315.1
Klebsiella oxytoca strain LMG 3055[T]	JX424964.1	JX425093.1	JX425223.1	JX425349.1

Klebsiella pneumoniae subsp. *ozaenae* strain LMG 3113^{T}	JX424965.1	JX425094.1	JX425224.1	JX425350.1
Klebsiella pneumoniae subsp. pneumoniae strain LMG 2095^{T}	JX424966.1	JX425095.1	JX425225.1	JX425351.1
Klebsiella pneumoniae subsp. *rhinoscleromatis* strain LMG 3184^{T}	JX424967.1	JX425096.1	JX425226.1	JX425352.1
Klebsiella singaporensis strain LMG 23571^{T}	JX424968.1	JX425097.1	JX425227.1	JX425353.1
Kluyvera ascorbata strain LMG 7871^{T}	JX424974.1	JX425103.1	JX425233.1	JX425359.1
Kluyvera cryocrescens strain LMG 7859^{T}	JX424975.1	JX425104.1	JX425234.1	JX425360.1
Kluyvera intermedia strain LMG 2785^{T}	JX424976.1	JX425105.1	JX425235.1	JX425361.1
Kosakonia arachidis strain KCTC 22375^{T}	JX424888.1	JX425018.1	JX425147.1	JX425274.1
Kosakonia cowanii strain BCC 008	JX424883.1	JX425013.1	JX425142.1	EU629165
Kosakonia cowanii strain BCC 009	JX424884.1	JX425014.1	JX425143.1	EU629166
Kosakonia cowanii strain BCC 011	JX424885.1	JX425015.1	JX425144.1	EU629167
Kosakonia cowanii strain LMG 23569^{T}	JX424882.1	JX425012.1	JX425141.1	JX425271.1
Kosakonia oryzae strain LMG 24251^{T}	JX424886.1	JX425016.1	JX425145.1	JX425272.1
Kosakonia oryzendophytica strain LMG 26432^{T}	KP345915.1	KP345907.1	KP345911.1	KP345904.1
Kosakonia oryziphila strain LMG 26429^{T}	LN873150.1	LN873151.1	LN873152.1	LN873153.1
Kosakonia pseudosacchari strain JM-387^{T}	KP345914.1	KP345906.1	KP345910.1	KP345903.1
Kosakonia quasisacchari strain WCHEs120001^{T}	MK613095.1	MK613096.1	MK613097.1	MK613098.1
Kosakonia radicincitans strain LMG 23767^{T}	JX424887.1	JX425017.1	JX425146.1	JX425273.1
Kosakonia sacchari strain LMG 26783^{T}	HG931156.1	HG931162.1	HG931168.1	JQ001786.1
Kosakonia sacchari strain LMG 26784	HG931157.1	HG931163.1	HG931169.1	JQ001788.1
Kosakonia sacchari strain LMG 26785	HG931158.1	HG931164.1	HG931170.1	JQ001789.1
Kosakonia sacchari strain LMG 26786	HG931159.1	HG931165.1	HG931171.1	JQ001787.1
Kosakonia sacchari strain LMG 26787	HG931160.1	HG931166.1	HG931172.1	JQ001790.1
Leclercia adecarboxylata strain LMG 2803^{T}	JX424867.1	JX424997.1	JX425126.1	JX425256.1
Lelliottia amnigena strain LMG 2784^{T}	JX424870.1	JX425000.1	JX425129.1	JX425259.1
Lelliottia amnigena strain LMG 3000	JX424871.1	JX425001.1	JX425130.1	JX425260.1
Lelliottia amnigena strain LMG 3004	JX424872.1	JX425002.1	JX425131.1	JX425261.1
Lelliottia amnigena strain LMG 3006	JX424873.1	JX425003.1	JX425132.1	JX425262.1
Lelliottia nimipressuralis strain LMG 10245^{T}	JX424868.1	JX424998.1	JX425127.1	JX425257.1
Lelliottia nimipressuralis strain NCPPB 440	JX424869.1	JX424999.1	JX425128.1	JX425258.1
Mangrovibacter plantisponsor strain LMG 24236	JX424958.1	JX425087.1	JX425217.1	JX425343.1
Pluralibacter gergoviae strain CCUG 21193	JX424875.1	JX425005.1	JX425134.1	JX425264.1
Pluralibacter gergoviae strain CCUG 29886	JX424876.1	JX425006.1	JX425135.1	JX425265.1
Pluralibacter gergoviae strain CCUG 53235	JX424877.1	JX425007.1	JX425136.1	JX425266.1
Pluralibacter gergoviae strain LMG 5739^{T}	JX424874.1	JX425004.1	JX425133.1	JX425263.1
Pluralibacter pyrinus strain KCTC 2586	JX424879.1	JX425009.1	JX425138.1	JX425268.1
Pluralibacter pyrinus strain KCTC 2588	JX424880.1	JX425010.1	JX425139.1	JX425269.1
Pluralibacter pyrinus strain KCTC 2590	JX424881.1	JX425011.1	JX425140.1	JX425270.1
Pluralibacter pyrinus strain LMG 22970^{T}	JX424878.1	JX425008.1	JX425137.1	JX425267.1
Raoultella planticola strain LMG 7870^{T}	JX424972.1	JX425101.1	JX425231.1	JX425357.1
Raoultella terrigena strain LMG 3222^{T}	JX424973.1	JX425102.1	JX425232.1	JX425358.1

Serratia entomophila strain ICMP 8760	JX424927.1	JX425057.1	JX425186.1	JX425313.1
Serratia entomophila strain ICMP 8764	JX424928.1	JX425058.1	JX425187.1	JX425314.1
Serratia entomophila strain ICMP 8767	JX424929.1	JX425059.1	JX425188.1	JX425315.1
Serratia entomophila strain LMG 8456^T	JX424926.1	JX425056.1	JX425185.1	JX425312.1
Serratia ficaria strain CCM 3419	JX424915.1	JX425045.1	JX425174.1	JX425301.1
Serratia ficaria strain CCM 3420	JX424916.1	JX425046.1	JX425175.1	JX425302.1
Serratia ficaria strain CCUG 20935	JX424917.1	JX425047.1	JX425176.1	JX425303.1
Serratia ficaria strain LMG 7881^T	JX424914.1	JX425044.1	JX425173.1	JX425300.1
Serratia fonticola strain CCM 3408	JX424919.1	JX425049.1	JX425178.1	JX425305.1
Serratia fonticola strain LMG 7882^T	JX424918.1	JX425048.1	JX425177.1	JX425304.1
Serratia glossinae DSM 22080 strain CCUG 57457^T	JX424948.1	JX425078.1	JX425207.1	JX425334.1
Serratia glossinae strain CCUG 31137	JX424920.1	JX425050.1	JX425179.1	JX425306.1
Serratia grimesii strain CCM 2718	JX424922.1	JX425052.1	JX425181.1	JX425308.1
Serratia grimesii strain LMG 7883^T	JX424921.1	JX425051.1	JX425180.1	JX425307.1
Serratia liquefaciens strain CCM 2716	JX424950.1	JX425080.1	JX425209.1	JX425336.1
Serratia liquefaciens strain DSM 30064	JX424951.1	JX425081.1	JX425210.1	JX425337.1
Serratia liquefaciens strain DSM 30066	JX424952.1	JX425082.1	JX425211.1	JX425338.1
Serratia liquefaciens strain LMG 7884^T	JX424949.1	JX425079.1	JX425208.1	JX425335.1
Serratia marcescens subsp. *marcescens* strain LMG 2792^T	JX424930.1	JX425060.1	JX425189.1	JX425316.1
Serratia marcescens subsp. *marcescens* strain LMG 3271	JX424931.1	JX425061.1	JX425190.1	JX425317.1
Serratia marcescens subsp. *sakuensis* strain CCM 7122^T	JX424932.1	JX425062.1	JX425191.1	JX425318.1
Serratia nematodiphila strain DSM 21420^T	JX424953.1	JX425083.1	JX425212.1	JX425339.1
Serratia odorifera strain CCUG 32567	JX424936.1	JX425066.1	JX425195.1	JX425322.1
Serratia odorifera strain CCUG 33344	JX424937.1	JX425067.1	JX425196.1	JX425323.1
Serratia odorifera strain LMG 25869	JX424938.1	JX425068.1	JX425197.1	JX425324.1
Serratia odorifera strain LMG 7885^T	JX424935.1	JX425065.1	JX425194.1	JX425321.1
Serratia plymuthica strain LMG 6823	JX424945.1	JX425075.1	JX425204.1	JX425331.1
Serratia plymuthica strain LMG 6825	JX424946.1	JX425076.1	JX425205.1	JX425332.1
Serratia plymuthica strain LMG 6827	JX424947.1	JX425077.1	JX425206.1	JX425333.1
Serratia plymuthica strain LMG 7886	JX424944.1	JX425074.1	JX425203.1	JX425330.1
Serratia proteamaculans strain CCUG 51551	JX424934.1	JX425064.1	JX425193.1	JX425320.1
Serratia proteamaculans strain LMG 8751^T	JX424933.1	JX425063.1	JX425192.1	JX425319.1
Serratia quinivorans strain CCM 4705	JX424924.1	JX425054.1	JX425183.1	JX425310.1
Serratia quinivorans strain CCM 4745	JX424925.1	JX425055.1	JX425184.1	JX425311.1
Serratia quinivorans strain LMG 7887^T	JX424923.1	JX425053.1	JX425182.1	JX425309.1
Serratia rubidaea strain LMG 5019^T	JX424939.1	JX425069.1	JX425198.1	JX425325.1
Serratia rubidaea strain LMG 7804	JX424940.1	JX425070.1	JX425199.1	JX425326.1
Serratia rubidaea strain LMG 7997	JX424941.1	JX425071.1	JX425200.1	JX425327.1
Serratia rubidaea strain LMG 7999	JX424942.1	JX425072.1	JX425201.1	JX425328.1
Serratia ureilytica strain LMG 28860^T	JX424943.1	JX425073.1	JX425202.1	JX425329.1

Xenorhabdus nematophila strain ATCC 19061^T	FN667742.1	FN667742.1	FN667742.1	FN667742.1
Yokenella regensburgei strain LMG 7872^T	JX424959.1	JX425088.1	JX425218.1	JX425344.1

Table S3. The GenBank/EMBL/DDBJ accession numbers for genome sequences used in this study.

**Strains**	**Accession Number**
Kosakonia arachidis KCTC 22375^T	GCA_900116535.1
Kosakonia cowanii LMG 23569^T	CP019445.1
Kosakonia oryzae LMG 24251^T	NZ_CP014007.2
Kosakonia oryzendophytica LMG 26432^T	GCA_900094925.1
Kosakonia oryziphila LMG 26429^T	GCA_900094795.1
Kosakonia pseudosacchari JM-387^T	GCA_900184035.1
Kosakonia quasisacchari strain WCHEs120001^T	GCA_004331415.1
Kosakonia radicincitans LMG 23767^T	CP018016.1
Kosakonia sacchari LMG 26783^T	CP007215.3

Supplementary Table S4. *atpD*, *gyrB* and *infB*, *rpoB* gene sequence similarities between strain FY-07 and type strains of *Kosakonia* and other phylogenetically related species.

**Strains**	**Gene Sequence Similarity (%) with Strain FY-07**			
	atpD	***gyrB***	***infB***	***rpoB***
Kosakonia oryzendophytica LMG 26432^T	95.02%	98.65%	99.19%	99.37%
Kosakonia pseudosacchari JM-387^T	92.21%	89.49%	89.92%	92.94%
Kosakonia sacchari LMG 2678^T	92.68%	88.95%	89.92%	91.68%
Kosakonia quasisacchari WCHEs120001^T	91.59%	88.81%	88.94%	93.25%
Kosakonia radicincitans LMG 23767^T	88.79%	89.08%	89.11%	93.88%
Kosakonia oryzae LMG 24251^T	89.25%	88.01%	88.46%	94.03%
Kosakonia cowanii LMG 23569^T	90.81%	89.76%	87.48%	90.89%
Kosakonia arachidis KCTC 22375^T	89.25%	86.39%	88.29%	92.94%
Kosakonia oryziphila LMG 26429^T	88.79%	87.74%	87.97%	91.99%
Enterobacter ludwigii LMG 23768^T	89.72%	86.52%	86.50%	92.46%
Enterobacter asburiae DSM 17506^T	89.72%	87.20%	84.23%	91.84%

Table S5. Pairwise similarities of concatenated partial *atpD*, *gyrB*, *infB* and *rpoB* gene sequences for type strains of the genus *Kosakonia*.

**Strains**	**Gene Sequence Similarity (%) with Strain FY-07**
	**MLSA**
Kosakonia oryzendophytica LMG 26432^T	98.07%
Kosakonia pseudosacchari JM-387^T	91.08%
Kosakonia sacchari LMG 26783^T	90.74%
Kosakonia quasisacchari WCHEs120001^T	90.59%
Kosakonia radicincitans LMG 23767^T	90.17%

Kosakonia oryzae LMG 24251^T	89.87%
Kosakonia cowanii LMG 23569^T	89.76%
Kosakonia arachidis KCTC 22375^T	89.11%
Kosakonia oryziphila LMG 26429T^T	89.07%

Table S6. The difference of G+Ccontent between FY-07 and type strains of the genus *Kosakonia*.

**Strains**	**G+C Content Difference with Strain FY-07 (%)**
Kosakonia oryzendophytica LMG 26432^T	0.11
Kosakonia sacchari LMG 26783^T	0.12
Kosakonia pseudosacchari JM-387^T	0.28
Kosakonia oryzae LMG 24251^T	0.39
Kosakonia radicincitans LMG 23767^T	0.06
Kosakonia quasisacchari WCHEs120001^T	0.28
Kosakonia oryziphila LMG 26429^T	0.87
Kosakonia arachidis KCTC 22375^T	1.08
Kosakonia cowanii LMG 23569^T	2.55
